# Femtograms of Interferon-γ Suffice to Modulate the Behavior of Jurkat Cells: A New Light in Immunomodulation

**DOI:** 10.3390/ijms18122715

**Published:** 2017-12-15

**Authors:** Sara Castiglioni, Vincenzo Miranda, Alessandra Cazzaniga, Marilena Campanella, Michele Nichelatti, Marco Andena, Jeanette A. M. Maier

**Affiliations:** 1 Dipartimento di Scienze Biomediche e Cliniche L. Sacco, Università di Milano, I-20157 Milan, Italy; alessandra.cazzaniga@unimi.it (A.C.); marcoandena@gmail.com (M.A.); jeanette.maier@unimi.it (J.A.M.M.); 2 Clinical Research Unit, GUNA S.p.a., Via Palmanova, 71, 20132 Milan, Italy; v.miranda@guna.it (V.M.); m.campanella@guna.it (M.C.); 3 Service of Biostatistics Hematology Department Niguarda Ca’ Granda Hospital, 20162 Milan, Italy; michele_nichelatti@hotmail.com

**Keywords:** interferon-γ, Jurkat cells, immunomodulation, STAT-1, SOCS-1

## Abstract

Since interferon-γ (IFN-γ) tunes both innate and adaptive immune systems, it was expected to enter clinical practice as an immunomodulatory drug. However, the use of IFN-γ has been limited by its dose-dependent side effects. Low-dose medicine, which is emerging as a novel strategy to treat diseases, might circumvent this restriction. Several clinical studies have proved the efficacy of therapies with a low dose of cytokines subjected to kinetic activation, while no in vitro data are available. To fill this gap, we investigated whether low concentrations, in the femtogram range, of kinetically activated IFN-γ modulate the behavior of Jurkat cells, a widely used experimental model that has importantly contributed to the present knowledge about T cell signaling. In parallel, IFN-γ in the nanogram range was used and shown to activate Signal transducer and activator of transcription (STAT)-1 and then to induce suppressor of cytokine signaling-1 (SOCS-1), which inhibits downstream signaling. When added together, femtograms of IFN-γ interfere with the transduction cascade activated by nanograms of IFN-γ by prolonging the activation of STAT-1 through the downregulation of SOCS-1. We conclude that femtograms of IFN-γ exert an immunomodulatory action in Jurkat cells.

## 1. Introduction

Interferon-γ (IFN-γ), a pleotropic cytokine secreted mainly by T, NK, and TNK cells after the activation of the immune system by virus, intracellular bacteria, and transplantation [1], is fundamental to heighten the defense against infections and tumors. IFN-γ activates cells of the innate immune system and plays a role in the development of Th1 lymphocytes and regulatory T cells, while it inhibits the differentiation of Th2 cells [2]. It is crucial to maintain the homeostasis of the host and finely tunes the innate and adaptive immune systems. Because of its relevant immunomodulatory functions, IFN-γ has inspired clinical applications in a variety of diseases, including tubercolosis, fungal and viral infections, cystic fibrosis, and chronic granulomatous disease [1]. Since IFN-γ also exerts antifibrotic effects, it has been used in chronic hepatitis and in idiopathic pulmonary fibrosis, but unsuccessfully [1]. In cancer, the scenario is rather complicated because IFN-γ is involved in tumor immune surveillance and is cytostatic, but it also has protumorigenic effects [2], and clinical trials have yielded discouraging results [1]. Besides its role in immunity, IFN-γ also influences the central nervous system. Interestingly, IFN-γ modulates sleep patterns by altering the ascending reticular activating system [3]. In humans, IFN-γ promotes N-REM sleep [4] and low amounts of IFN-γ correlate with difficulty in maintaining normal sleep [3].

In general, the clinical applications of IFN-γ are limited by its dose-dependent side effects characterized by massive signs of inflammation [1]. Moreover, high levels of IFN-γ in the absence of any stimuli have detrimental effects and contribute to the pathogenesis of some autoimmune diseases [5].

IFN-γ signals by binding a multi-chain receptor composed of two IFN-γR1 subunits and IFN-γR2 chains. IFN-γR1 binds its ligand and, together with IFN-γR2, initiates the signal transduction cascade that culminates with the activation of the JAK/STAT-1 pathway [6]. Upon translocation to the nucleus, STAT-1 binds to promoter elements and modulates the transcription of IFN-γ-sensitive genes, among which interferon regulatory factor-1 (IRF-1). IRF-1 then contributes to the activation of suppressor of cytokine signaling-1 (SOCS-1), which inhibits downstream IFN-γ signaling [7,8]. 

In spite of its important immunomodulatory actions, IFN-γ is hard to handle in clinics because of its adverse effects [1]. An interesting approach to limit side effects is offered by low-dose medicine, i.e., the use of molecules in the femtogram/mL (fg/mL) range [9], wherein the physiological concentrations of messenger molecules, such as cytokines and growth factors, reside in vivo [10]. Since disease can be viewed as an imbalance in intercellular signaling, low-dose medicine can restore homeostasis by harmonizing signaling operated by various cytokines [11]. Some studies have demonstrated the efficacy and the lack of side effects of low-dose cytokines subjected to sequential kinetic activation (SKA). SKA is a pharmaceutical technology, which consists of shaking a solution to potentiate the pharmacological action of low-dose compounds and obtain detectable results with concentrations below those generating side effects. Indeed, both clinical and preclinical studies indicate that SKA cytokines induce biological responses. In patients with moderate psoriasis, the simultaneous oral administration of low doses of SKA IL-4, IL-10, and IL-11 reduces the severity and the extent of the lesions [12]. Similarly, in patients with rheumatoid arthritis, oral therapy with low doses of SKA IL-4, IL-10, and anti IL-1 antibodies is a promising tool for the management of disease remission obtained with biological agents [13].

In one study, low doses of SKA IL-12 and IFN-γ, co-delivered via oral route, were found to inhibit bronchial hyper-reactivity in a murine model of allergic asthma by balancing Th1/Th2 and restoring normal cytokine levels [14]. This study was the first to provide convincing evidence of the importance of mechanically activated cytokine solutions, which show a therapeutic efficacy in the femtogram range that is not detectable with non-activated cytokine solutions at the same concentrations [14]. Additional studies ex vivo and in vitro have confirmed that low concentrations of SKA cytokines are active. In vitro, exposure to low doses of SKA cytokines reduced oxidative stress in keratinocytes from vitiligo skin [15]. In addition, low doses of SKA IL-12 have been found to increase the lytic activity of T lymphocytes on cells from patients with lung cancer and inhibit the proliferation of lung carcinoma cells ex vivo [16]. Interestingly, low doses of SKA IFN-γ have been found to enhance the ex vivo cytotoxic activity of NK cells from patients with early-stage colon cancer [17], thus suggesting that low doses of SKA IFN-γ have an immunomodulatory effect. The same authors then demonstrated that consecutive exposure to low doses of SKA IL-4 and IL-12 enhances the immunostimulatory functions of dendritic cells from early-stage colon cancer patients [18].

No data are available in vitro on the effects of SKA IFN-γ. Since IFN-γ plays a critical role in T-cell immunity, we performed experiments on Jurkat cells, a human leukemia T-cell line widely accepted as a valid experimental model. Indeed, Jurkat cells have allowed major advances in our understanding of signal transduction in T-cells [19]. For this reason, we argued that Jurkat cells might represent an ideal model for investigating the effects of SKA IFN-γ in the fg/mL range (fg SKA IFN-γ). In parallel, recombinant (r) IFN-γ was utilized in the classical nanogram range. Our principal finding is that fg SKA IFN-γ interferes with IFN-γ signaling.

## 2. Results

### 2.1. The Response of Jurkat Cells to IFN-γ

**Figure 1:**
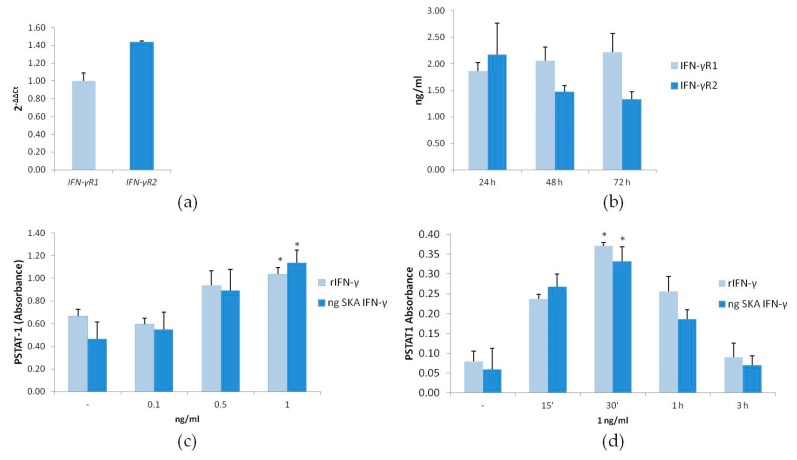
IFN-γR levels under basal culture conditions and STAT-1 activation in response to rIFN-γ and SKA IFN-γ in the ng/mL range (ng SKA IFN-γ). (**a**) After 24 h in culture, Jurkat cells were analyzed for *IFN-γR1* and *IFN-γR2* transcripts by Real-Time PCR and (**b**) for IFN-γR1 and IFN-γR2 protein level by ELISA after 24, 48, and 72 h of culture. (**c**,**d**) The phosphorylation of STAT-1 (PSTAT-1) was evaluated on Jurkat cells after treatment with different doses of rIFN-γ or ng SKA IFN-γ (0.1, 0.5, and 1 ng/mL) for 1 h (**c**) or with 1 ng/mL of rIFN-γ or ng SKA IFN-γ for different times (15 min, 30 min, 1 h, and 3 h). The results are the mean of three experiments in triplicates. -: non-treated cells. * *p* < 0.05.

Since both IFN-γR1 and -R2 are necessary to transduce intracellular signaling, we evaluated the expression of their transcripts by Real Time PCR. As shown in Figure 1a, under basal culture conditions, Jurkat cells express these two chains. We then analyzed the levels of the proteins by ELISA after 24, 48, and 72 h in culture and found that the amounts of IFN-γR1 and -R2 remain steady at these time points (Figure 1b).

IFN-γ utilizes predominantly the JAK/STAT-1 pathway for signal transduction [6]. Since STAT-1 is activated when it is phosphorylated by JAK, we measured the phosphorylation of STAT-1 by ELISA in cells exposed for 60 min to different concentrations of rIFN-γ or SKA IFN-γ in the ng/mL range (ng SKA IFN-γ). We found a significant induction of phosphorylated STAT-1 (PSTAT-1) with 1 ng/mL of the cytokine (Figure 1c). This concentration was selected for further studies. Jurkat cells were exposed to rIFN-γ or ng SKA IFN-γ (1 ng/mL) for different times. STAT-1 phosphorylation peaked after 30 min and returned to basal levels after 3 h (Figure 1d). These results demonstrate that ng SKA IFN-γ and rIFN-γ exert similar effects and activate STAT-1.

### 2.2. The Response of Jurkat Cells to Low Doses of SKA IFN-γ: IFN-γR1 and -R2 and STAT-1 Phosphorylation

**Figure 2:**
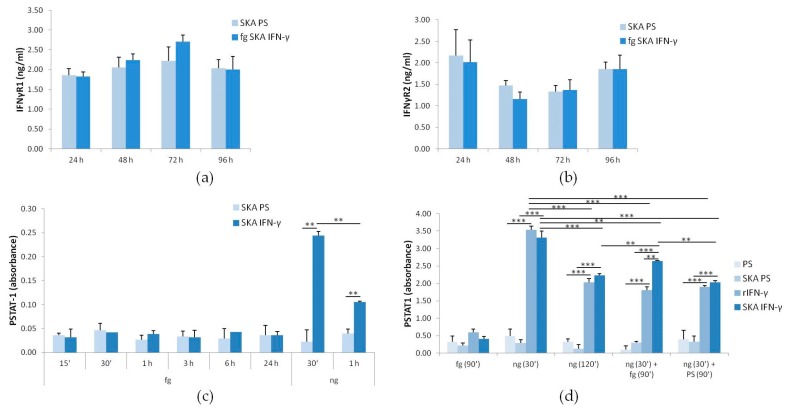
The response of Jurkat cells to fg SKA IFN-γ. (**a**,**b**) Jurkat cells were treated for 24, 48, 72, and 96 h with fg SKA IFN-γ (10 fg/mL) or SKA PS as a control. IFN-γR1 and IFN-γR2 levels were measured by ELISA. (**c**) Jurkat cells were treated with fg SKA IFN-γ (10 fg/mL) for different times or with ng SKA IFN-γ (1 ng/mL) for 30 min and 1 h as positive controls. PSTAT-1 ELISA was performed on cell lysates. Control cells were treated with SKA physiological solution (SKA PS). (**d**) Jurkat cells were treated (i) with fg SKA IFN-γ (10 fg/mL) for 90 min; (ii,iii) with ng SKA IFN-γ (1 ng/mL) for 30 and 120 min; (iv) with ng SKA IFN-γ for the initial 30 min and then with fg SKA IFN-γ or with 10 fg rIFN-γ for the following 90 min; (v) with ng SKA IFN-γ for the initial 30 min and then with SKA PS or PS for the following 90 min. Controls were treated with SKA PS or PS only. ELISA was performed on cell lysates. The results are the mean of three experiments in triplicates. ** *p* < 0.01, *** *p* < 0.001.

We then evaluated the effect of low doses of SKA IFN-γ (fg SKA IFN-γ). By ELISA, we did not observe any significant alteration of the total amounts of IFN-γR1 and -R2 in Jurkat cells treated with fg SKA IFN-γ (10 fg/mL) for 24, 48, 72, and 96 h vs. their controls exposed to SKA Physiological Solution alone (SKA PS, the same amount used for fg SKA IFN-γ) (Figure 2a,b). 

While STAT-1 is rapidly and transiently phosphorylated by nanograms of IFN-γ [7,8], we hypothesized that fg SKA IFN-γ might require longer times to exert its action. Therefore, we analyzed the phosphorylation of STAT-1 in Jurkat cells treated with fg SKA IFN-γ for various times from 15 min to 24 h. While ng SKA IFN-γ (1 ng/mL) markedly induced STAT-1 phosphorylation, fg SKA IFN-γ showed no effect at any time tested (Figure 2c). 

The possibility that fg SKA IFN-γ might interfere with IFN-γ was then evaluated. The cells were treated as follows: (i) with fg SKA IFN-γ for 90 min; (ii,iii) with ng SKA IFN-γ (1 ng/mL) for 30 or 120 min; (iv) with ng SKA IFN-γ (1 ng/mL) for the initial 30 min and, after the cells were washed, exposed to either rIFN-γ or SKA IFN-γ (both 10 fg/mL) for the following 90 min; (v) with ng SKA IFN-γ (1 ng/mL) for the initial 30 min and, after the cells were washed, exposed to physiological solution as controls (PS and SKA PS) for the following 90 min. As expected, ng SKA-IFN-γ and rIFN-γ induced STAT-1 after 30 min (an ~10-fold increase compared with the control) and declined thereafter (an ~4-fold induction) (Figure 2d). Surprisingly, the addition of fg SKA-IFN-γ after the treatment with ng SKA IFN-γ sustained STAT-1 activation, while 10 fg of rIFN-γ exerted no effect (Figure 2d). These results indicate that fg SKA IFN-γ contributes to the regulation of IFN-γ signaling. 

### 2.3. The Response of Jurkat Cells to Low Doses of SKA IFN-γ: Expression of SOCS-1

**Figure 3:**
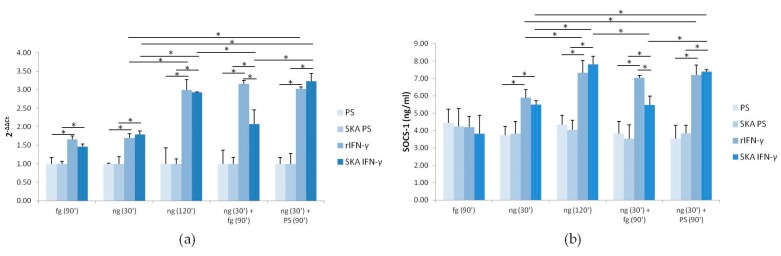
SOCS-1 in Jurkat cells in response to fg SKA IFN-γ. (**a**) Jurkat cells were treated as described in Figure 2d. *SOCS-1* RNA was analyzed by real-time PCR. (**b**) Cells treated as in (**a**) were evaluated for SOCS-1 levels by ELISA. The results are the mean of three experiments in triplicates. * *p* < 0.05.

SOCS-1 is fundamental for limiting IFN-γ action [8,20]. We treated the cells as described in Figure 2c and evaluated *SOCS-1* levels by Real Time PCR. We found that 90 min of exposure to fg SKA IFN-γ significantly upregulated *SOCS-1* transcript (Figure 3a). In agreement with previous reports [20], ng SKA IFN-γ induced *SOCS-1* expression after 30 and 120 min. In cells exposed to ng SKA IFN-γ for 30 min and then to fg SKA IFN-γ for additional 90 min, *SOCS-1* transcript was lower than in cells treated with ng SKA IFN-γ for 120 min, while this effect was not detected in cells exposed to 10 fg of rIFN-γ. SKA PS and PS do not impact on *SOCS-1* expression. We then measured the protein levels of SOCS-1 by ELISA assay in Jurkat cells treated as described above. We did not detect any increase of SOCS-1 in cells treated with fg SKA IFN-γ alone (Figure 3b), but fg SKA IFN-γ attenuated the increase of SOCS-1 after exposure to ng SKA IFN-γ. On the contrary, 10 fg of rIFN-γ had no effect. These results demonstrate that fg SKA IFN-γ interferes with the modulation of the levels of SOCS-1.

## 3. Discussion

IFN-γ exerts crucial immunostimulatory and immunomodulatory actions, since it regulates immune system development, maturation, and function [1]. Because of this, IFN-γ has been used as an adjuvant for vaccine therapies and chemotherapies, in the treatment of some autoimmune diseases and tubercolosis [1]. However, the results were often modest and eventually associated with side effects mainly due to an overwhelming inflammatory response [1]. It is probably necessary to tune the use of the cytokine in the unique setting of the different diseases in different individuals.

Recently, several studies have suggested that low-dose therapy might become a new option to manage diseases. In this light, the paradigm “the more the better” has been revisited. Metronomic chemotherapy is an example. Low doses of various chemotherapeutic drugs are chronically administered in cancer patients with antitumor efficacy and minimal toxicity. The effectiveness is due to the inhibition of angiogenesis, the restoration of anticancer immune response and the induction of tumor dormancy [21]. It can be concluded that low-dose chemotherapy finely shapes various events that cooperate in keeping tumors under control. 

Similarly, the use of low doses of cytokines, neurotransmitters, and hormones has recently been shown to control and harmonize cellular functions in order to restore the original homeostatic physiological condition. In a recent study, Uberti et al. demonstrate that the activated formulations of acetylcholine increase keratinocyte viability, proliferation, and migration and decrease the production of reactive oxygen species and oxygen consumption [22]. This study also confirms previous results [14] about the relevance of the mechanical activation of the solutions to potentiate the pharmacological action of low-dose compounds. A low dose of kinetically activated cytokines was effective in balancing the immune response in diseases associated with immune dysfunction, as demonstrated in patients with psoriasis, rheumatoid arthritis, and chronic childhood eczema [12,13,23]. Low doses of SKA IL-12 (10 fg/mL) increased the number of CD4 and CD8 T lymphocytes and inhibited T-regulatory cells in cultures of peripheral blood mononuclear cells derived from non-small lung cancer patients [16]. As for IFN-γ, one study showed that low doses of SKA IFN-γ enhanced the activity of natural killer cells from patients with early-stage colon cancer [17], and another study showed that consecutive exposure to low doses of SKA IL-4 and IL-12 enhanced the immunostimulatory functions of dendritic cells from similarly afflicted patients [18].

It is noteworthy that these low doses of cytokines exert their activity only after being kinetically activated [12,18,23]. The mechanisms involved the potentiation of the pharmacological action of low-dose compounds have not been elucidated and deserve more studies. It is feasible that mechanical forces applied by shaking modulate the structure of the molecule in a way that optimizes its binding with its cognate receptor. However, the very low concentrations used prevent the possibility of performing the classical biochemical studies to investigate the interaction with the receptor. 

Here we focused on the effects of low doses of SKA IFN-γ in a well established experimental model, i.e., Jurkat cells. We are aware of the limitations of long-term cell lines, but they provide a relatively simple tool to characterize intracellular pathways and do not present the problems due to individual variability occurring with primary cultures. In agreement with previous reports, we show that 1 ng/mL of IFN-γ activates STAT-1, which initiates the transcription of IFN-γ-dependent genes, among which is *SOCS-1*. SOCS-1 acts as a negative-feedback regulator of JAK signaling [24]. Indeed, SOCS-1 both shuts down JAK kinase activity and promotes its degradation [24]. IFN-γ induction of *SOCS-1* mRNA is accompanied by the upregulation of SOCS-1 at the protein level. As expected, after 120 min, the increase of SOCS-1 paralleled the decreased phosphorylation of STAT-1 (Figure 2d and Figure 3b). Additionally, fg SKA IFN-γ alone induces an increase of *SOCS-1* mRNA, without reaching the threshold to be translated into an increase of the protein. It is intriguing that the induction of *SOCS-1* mRNA by fg SKA IFN-γ occurs independently from the phosphorylation of STAT-1, thus suggesting that other signaling pathways are activated by fg SKA IFN-γ. As mentioned above, there are technical restraints that prevent the demonstration of the binding of fg SKA IFN-γ with IFN-γR1 and -R2. The only evidence we provide is that fg SKA IFN-γ does not impact the total amounts of the two receptors.

**Figure 4:**
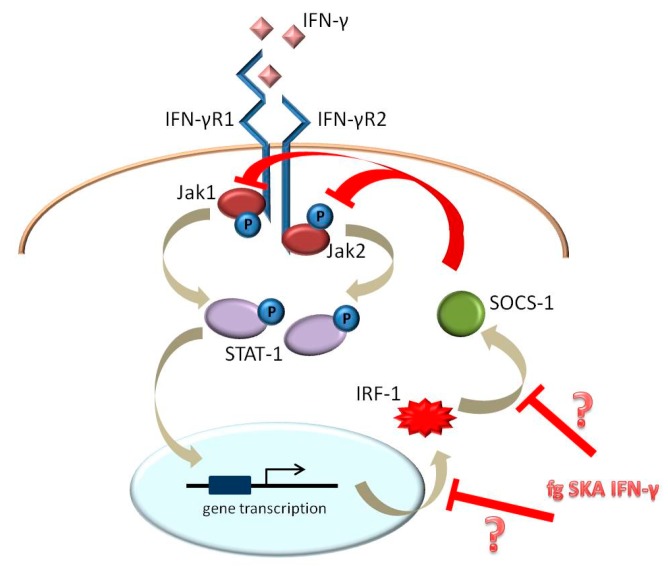
Illustration summarizing our present knowledge of the action of fg SKA IFN-γ. fg SKA IFN-γ might induce intracellular signals that interfere with *IRF-1* gene transcription or that reduce IRF-1-mediated SOCS-1 activation. This results in the inhibition of the IFN-γ-induced negative feedback, thus extending the duration of its action. ├ means inhibition.

Unexpectedly, we observed that, when fg SKA IFN-γ is added after 1 ng/mL of IFN-γ, it attenuates the increase of *SOCS-1* mRNA and protein induced by IFN-γ so that the amounts of SOCS-1 do not reach the threshold necessary to inhibit JAK-mediated STAT-1 phosphorylation. Consequently, fg SKA IFN-γ prolongs STAT-1 phosphorylation. We hypothesize that fg SKA IFN-γ induces intracellular signals that interfere with IFN-γ-induced negative feedback, thus extending the duration of its action (Figure 4). More studies are necessary to obtain a broader picture of the effects of fg SKA IFN-γ. In particular, it will be interesting to study the effects of chronic, repeated exposure of Jurkat cells to fg SKA IFN-γ to provide insights for clinical application. Indeed, a low dose of SKA IFN-γ is administered to patients for weeks and even months with the aim of restoring the homeostatic balance among various populations of lymphocytes. It will also be relevant to focus our attention on other regulatory proteins involved in controlling IFN-γ action. 

In conclusion, this is the first report showing that a low dose of SKA IFN-γ modulates signal transduction and gene expression in a well-established in vitro experimental model, thus paving the way to novel approaches in immunomodulation.

## 4. Materials and Methods

### 4.1. Cell Culture

Jurkat cells, purchased from ATCC, were cultured in RPMI containing 10% fetal bovine serum and 2 mM glutamine, at 37 °C and 5% CO_2_. All culture reagents used were from Euroclone. Before each treatment, 1 × 10^7^ cells were starved over night in RPMI containing 0.1% fetal bovine serum and then with ng SKA IFN-γ or fg SKA IFN-γ (1 ng/mL and 10 fg/mL, respectively) (GUNA S.p.a., Milan, Italy) or SKA Physiological Solution (SKA PS, GUNA S.p.a., Milan, Italy) as control. rIFN-γ (PromoKine, Catalog Number: C-60724) was also used in initial experiments and compared to the same concentrations of ng SKA IFN-γ. 

### 4.2. SKA Procedure

SKA IFN-γ and SKA PS were prepared by GUNA Laboratories (GUNA S.p.a., Milan, Italy) using a standardized method [12]. IFN-γ and PS were subjected to a shaking procedure consisting of vertical shaking with a 10 cm motion range. Shaking speed corresponds to 100 oscillations in 10 s. 

### 4.3. Real-Time PCR

Total RNA was extracted by the PureLink RNA Mini kit (Thermo Fisher Scientific, Monza, Italy; Catalog Number: 12183018A). Single-stranded cDNA was synthesized using the High Capacity cDNA Reverse Transcription Kit (Thermo Fisher Scientific, Catalog Number: 4368814), and real-time PCR was performed using TaqMan Gene Expression Assays (Thermo Fisher Scientific): Hs00166223_m1 (*IFNγR1*), Hs00985249_m1 (*IFNγR2*) and Hs00705164_s1 (*SOCS-1*). The housekeeping gene *GAPDH* (Hs99999905_m1) was used as an internal reference gene. Relative changes in gene expression were analyzed by the 2^−ΔΔ*C*t^ method. Experiments were performed twice in triplicate.

### 4.4. ELISA

For the quantitative determination of IFN-γR1 and IFN-γR2 in plasma membrane, Cusabio ELISA kit was used (Catalog Number: CSB-EL011051HU) according to the datasheet instructions. The PathScan PHOSPHO-STAT-1 (Tyr701) Sandwich ELISA Kit (Cell Signaling, Pero-MI, Italy; Catalog Number: 7234) was used to measure STAT-1 phosphorylation. Cell Lysis Buffer of Cell Signaling (Catalog Number: 9803) was used to lyse Jurkat cells under non-denaturing conditions and ELISA was performed according to the manufacturer’s instructions. SOCS-1 was measured using the Human SOCS-1 ELISA Kit of LSBio (Space Import-Export, Milano, Italy; Catalog Number: LS-F7983). Jurkat cells were lysed with three freeze–thaw cycles, and ELISA was performed according to the datasheet instructions. All ELISAs were performed at least three times, and each sample was measured in triplicate.

### 4.5. Statistical Analysis 

All data were validated and submitted to the usual descriptive analysis. One-way ANOVA and repeated measures ANOVA were carried out to verify the significance of between-samples and within-sample factors, as well as the group–factor interactions; the *p*-values deriving from multiple pairwise comparisons were corrected using the Scheffé and the Bonferroni methods, while the Levene’s test was used to assess the equality of error variances.
